# The Relevance of IL-1-Signaling in the Protection against Gram-Positive Bacteria

**DOI:** 10.3390/pathogens10020132

**Published:** 2021-01-28

**Authors:** Angelina Midiri, Giuseppe Mancuso, Concetta Beninati, Elisabetta Gerace, Carmelo Biondo

**Affiliations:** Department of Human Pathology, University of Messina, 90026 Messina, Italy; amidiri@unime.it (A.M.); mancusog@unime.it (G.M.); cbeninati@unime.it (C.B.); geraceelisabetta74@gmail.com (E.G.)

**Keywords:** IL-1R signaling, bacterial peritonitis models, neutrophil recruitment, inflammatory chemokines

## Abstract

Previous studies performed using a model of group B streptococcus (GBS)-induced peritoneal inflammation indicate that the interleukin-1 receptor (IL-1R) family plays an important role in the innate host defense against this encapsulated Gram-positive bacteria. Since the role of IL-1-dependent signaling in peritoneal infections induced by other Gram-positive bacteria is unknown, in the present study we sought to investigate the contribution of IL-1R signaling in host defenses against *Streptococcus pyogenes* (group A streptococcus or GAS) or *Staphylococcus aureus*, two frequent and global human Gram-positive extracellular pathogens. We analyzed here the outcome of GAS or *S. aureus* infection in IL-1R-deficient mice. After inoculated intraperitoneal (i.p.) inoculation with group A *Streptococcus* or *S. aureus*, all the wild-type (WT) control mice survived the challenge, while, respectively, 63% or 50% of IL-1-defective mice died. Lethality was due to the ability of both bacterial species to replicate and disseminate to the target organs of IL-1R-deficient mice. Moreover, the experimental results indicate that IL-1 signaling promotes the production of leukocyte attractant chemokines CXCL-1 and CXCL-2 and recruitment of neutrophils to bacterial infection sites. Accordingly, the reduced neutrophil recruitment in IL-1R-deficient mice was linked with decreased production of neutrophil chemokines. Collectively, our findings indicate that IL-1 signaling, as previously showed in host defense against GBS, plays a fundamental role also in controlling the progression and outcome of GAS or *S. aureus* disease.

## 1. Introduction

Gram-positive bacteria such as *Streptococcus pyogenes*, also known as the group A streptococci (GAS) and *Staphylococcus aureus* are among the most common human pathogens that cause a wide variety of diseases ranging from skin and soft tissue injuries to life-threatening illness [1,2,3,4,5]. An increase in the incidence of invasive infection caused by these two Gram-positive cocci has been reported in the past years [6,7,8,9]. The nasopharyngeal mucosa and associated lymphoid tissues are the most common sites of entry for GAS, which, however, may colonize and persist transiently in humans, often throughout childhood, without overt symptoms of disease [10,11]. Moreover, the nasopharyngeal mucosa is the primary reservoir for replication and dissemination of GAS not only between individuals but also to other sites of the body [12,13]. *Staphylococcus aureus*, is one of the most important nosocomial pathogens, responsible for serious infections, such as pneumonia, bloodstream infections, endocarditis peritonitis, septic arthritis, and osteomyelitis [3,8,14]. Around 20 to 30 percent of healthy individuals are persistently or transiently colonized by *S. aureus* that produces several virulence factors and activates different mechanisms to evade host immune defenses [14,15]. The IL-1 receptor family comprises ten receptors and accessory proteins that transduce signals coming from other IL-1 family members [16]. The IL-1R is the receptor that transduces the proinflammatory signal, while the IL-1RI is a decoy receptor, which binds IL-1 but is unable to initiate an intracellular response. The binding of IL-1α or IL-1β to IL-1R results in a structural change that binds the co-receptor IL-1R3 to IL-1R. The trimeric complex can then bind MyD88 that in turn triggers a selected group of kinases that through the activation of NFκB lead to the transcription of proinflammatory genes [16,17,18]. Since the IL-1 receptor family and Toll-like receptor (TLR) families share the cytoplasmic Toll-IL-1-Receptor (TIR) domain, essential inflammatory and innate immune responses are common to both families ligands [19]. IL-1 family has an important role in inflammation and activation of innate immune responses, potentiates the functional activity of phagocytes and promotes the expression of CXC chemokines and neutrophil recruitment [16,20,21,22]. Moreover, the complement system, a potent component of the innate immune response, has been shown to interact with the IL-1R signaling pathway in eliminating virus-containing cells [23].

Previous studies have shown that IL-1 signaling plays a crucial role in the restriction of invasive infection of different pathogens, including GAS, by driving neutrophil recruitment to the infection sites [12,24,25]. However, as recently reported, the role of IL-1 signaling in GAS infection is not completely known since neutrophil influx promoted by IL-1 signaling is host-protective in the skin and soft tissue but detrimental in the nasopharynx where this influx would promote instead GAS infection [25].

In light of this, it is important to define the contribution of the IL-1R signaling pathway to the pathogenesis of GAS infection in other sites of the body. Thus, in this study, we sought to define the contribution of the IL-1R signaling in the peritonitis caused by GAS. As with GAS also for S. aureus previous studies have investigated the role of IL-1 signaling in the outcome of *S. aureus* infection using different mouse models of infection including brain abscesses and septic arthritis. The results of these studies showed that bone-marrow-derived cells play key roles in most, but not all, experimental murine models of *S. aureus* infection where IL-1R-dependent neutrophil recruitment is fundamental on the outcome of *S. aureus* infection [26,27]. In light of the above, the mechanism used by this microorganism to counteract the activation by IL-1R-mediated innate immune responses is yet to be clarified [28,29,30,31].

In this study, in order to investigate the contribution of the IL-1R signaling pathway to the pathogenesis of GAS or *S. aureus* infection, we set up novel models of inflammation whereby we determined the number and types of cells present in the peritoneal cavity after injection of killed GAS or *S. aureus*. Moreover, using IL-1R-deficient mice, we demonstrated that the lack of IL-1R signaling was linked with decreased production of neutrophil chemokines and recruitment of neutrophils to GAS or *S. aureus* infection sites.

## 2. Results

### 2.1. IL-1R-Deficient Mice Are Susceptible to GAS or S. aureus Infection

**Figure 1:**
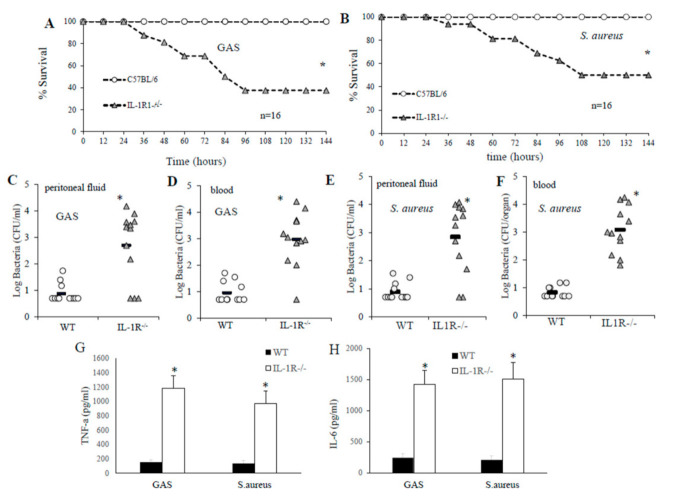
**Effect of interleukin-1 (IL-1) signaling on the outcome of the infection following an intraperitoneal (i.p.) challenge with group A streptococcus (GAS) or *S. aureus*.** Survival of wild-type (WT) and IL-1R-/- deficient mice after an i.p. challenge with 4 × 10^5^ colony-forming units (CFU) of GAS (A) or 5.5 × 10^5^ CFU of *S. aureus* (B). Mice were observed until 10 days after challenge, but further deaths did not occur. *, *p* < 0.05 versus WT mice, as determined by Kaplan–Meier survival plots. (C–F), Peritoneal lavage fluid and blood CFU counts of WT and IL-1R-deficient mice at 24 h after an i.p. challenge with 4 × 10^5^ CFU of GAS (C and D) or 5.5 × 10^5^ CFU of *S. aureus* (E,F). Log CFU was expressed as means ± SEM of five determinations, each conducted on a different animal, in the course of one experiment. Horizontal bars indicate mean values. (G,H), Plasma cytokine levels in WT and IL-1R-deficient mice at 24 h after an i.p. challenge with 4 × 10^5^ CFU of GAS or 5.5 × 10^5^ CFU of *S. aureus*. Cytokine levels are expressed as the means ± standard deviations of five determinations, each conducted with a different animal, during one experiment. *, *p* < 0.05 versus WT mice, by one-way analysis of variance and the Student-Keuls–Newman test.

To examine the contribution of IL-1 signaling in GAS or *S. aureus* infection we used two models of induced peritoneal inflammation. In preliminary experiments mice were inoculated intraperitoneal (i.p.) with doses of GAS or *S. aureus* strain, in order to identify a dose that resulted in no lethality in wild-type (WT) mice but capable of killing the IL-1R-deficient mice (data not shown). To investigate the role of IL-1signaling we initially tested IL-1R-deficient mice for susceptibility to GAS or *S. aureus* infection. After i.p. inoculation with 4 × 10^5^ colony-forming units (CFU) of GAS or 5.5 × 10^5^ CFU of *S. aureus*, all the WT control mice survived the challenge, while, respectively, 63% or 50% of IL-1-defective mice died (Figure 1, panel A and B). To further assess if the increased lethality was associated with an impaired ability of the host to control GAS or *S. aureus* growth, we performed colony counting in peritoneal lavage fluid and blood samples obtained at 24 h after i.p. challenge. Only rarely were CFU of GAS or *S. aureus* detected in samples obtained from WT animals while elevated CFU of both bacterial species was always present in samples obtained from IL-1R-deficient mice (Figure 1, C–F). In further experiments, we sought to ascertain whether cytokine levels were reduced in IL-1R-deficient mice inoculated with live GAS or *S. aureus*. To this end, plasma TNF-α and IL-6 protein levels were measured in WT and IL-1R-deficient mice at 24 h after a similar i.p. challenge with GAS or *S. aureus*. Elevate levels of these two cytokines were detected in IL-1R-defective animals compared to WT mice, as a consequence of the bacteremia in IL-1R-deficient mice (Figure 1 panels G and H).

### 2.2. IL-1 Signaling Is Required for Host Resistance against GAS and S. aureus

**Figure 2:**
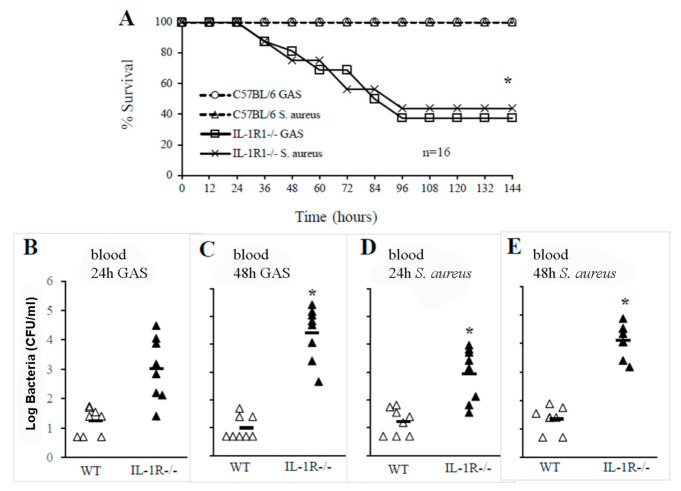
IL-1R signaling plays a crucial role in the clearance of GAS or *S. aureus in the blood*. (**A**), Survival of WT and IL-1R-/- mice after an i.v. challenge with 1 × 10^6^ CFU of GAS or 1.5 × 10^6^ CFU of *S. aureus*. Shown are the cumulative results of two experiments, each conducted with eight animals per group. *, *p* < 0.05 versus WT mice as determined with a Kaplan–Meier survival plot. Blood colony counts at 24 and 48 h after an i.v. challenge with 1 × 10^6^ CFU of GAS (**B**,**C**) or with 1.5 × 10^6^ CFU of *S. aureus* (**D**,**E**). Each determination was conducted with a different animal in the course of one experiment, involving eight animals per group. Horizontal bars indicate mean values. *, *p* < 0.05 versus WT mice, as determined by one-way analysis of variance and the Student–Keuls–Newman test.

In further experiments, we specifically investigated the involvement of IL-1 signaling in the clearance of GAS or *S. aureus* from the bloodstream. IL-IR-deficient mice and WT controls were infected with GAS or *S. aureus* via an i.v. route to mimic bacterial sepsis (Figure 2A). Under these conditions, approximately one-half of the IL-1R-deficient mice died after GAS challenge or developed neurological signs, including ataxia and paralysis whereas all WT mice survived. Similar effects of IL1-R deficiency were observed in a model of *S. aureus* sepsis (Figure 2A). Moreover, increased lethality was associated with severe bacteremia and higher colony counts in blood samples obtained from IL-1R-defective mice compared to those from WT mice at 24 and 48 h after infection (Figure 2, panels B–E). Thus, IL-1R signaling played a protective role not only against GAS, but also against *S. aureus*.

### 2.3. The Lack of IL-1 Signaling Has No Effects on Phagocytosis and Bactericidal Activity

In an attempt to find out whether the inability of IL-1R-deficient mice to control GAS or *S. aureus* infection could be ascribed to the decreased bactericidal activity of IL-1R-deficient mice compared to WT animals, bacteria were mixed with freshly drawn blood from WT or IL-1R-deficient mice and the bacteria were counted at different time points. However, as shown in Figure S1 and in agreement with previous results, obtained using GBS as a stimulus, GAS or S. aureus counts determined in IL-1R-deficient and WT blood cultures were not significantly different. Moreover, no differences in the phagocytosis and killing of these bacteria were found between WT and IL-1R-deficient bone marrow-derived macrophages (Figure S1).

### 2.4. IL-1 Signaling Is Required for the Production of the CXCL1/2 Chemokines

**Figure 3:**
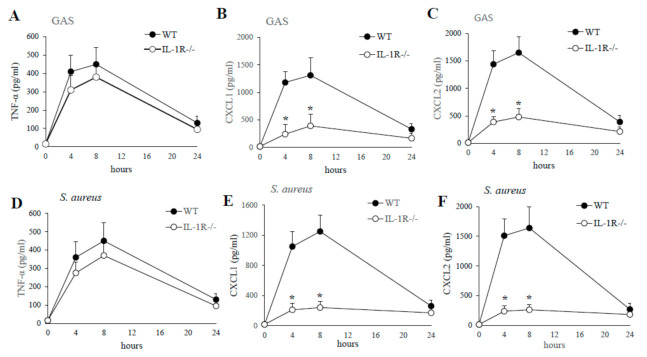
**Kinetics of cytokine production in peritoneal lavage fluid after a challenge with killed GAS or *S. aureus*.** TNF-α, CXCL1, CXCL2, protein levels were measured in peritoneal lavage fluid from WT and IL-1R-deficient mice, at the times indicated, after i.p. injection of killed GAS (0.5 mg; **A**–**C**) or *S. aureus* (0.5 mg; **D**–**F**). Data are expressed as means ± standard deviations of triplicate observations, each conducted with a different animal, in the course of a single experiment. *, *p* < 0.05 versus WT mice by one-way analysis of variance and the Student–Keuls–Newman test.

To determine the contribution of IL-1 signaling to GAS or *S. aureus* in vivo induced cytokine production, WT and IL-1R-deficient mice were injected with killed GAS or *S. aureus* and cytokine levels were measured in PFL samples collected at different times after stimulation. As shown in Figure 3, a significant decrease in the production of CXCL1/2 were detected in GAS or *S. aureus* stimulated PFL of IL-1R-deficient mice compared to WT mice already at four hours poststimulation. In contrast, no significant difference was detected in TNF-α levels between WT and IL-1R deficient mice (Figure 3).

To understand the role of the macrophages, in our i.p. model of infection, in chemokines production in response to GAS or *S. aureus* in further experiments we isolated resident peritoneal macrophages and stimulated them with live bacteria.

**Figure 4:**
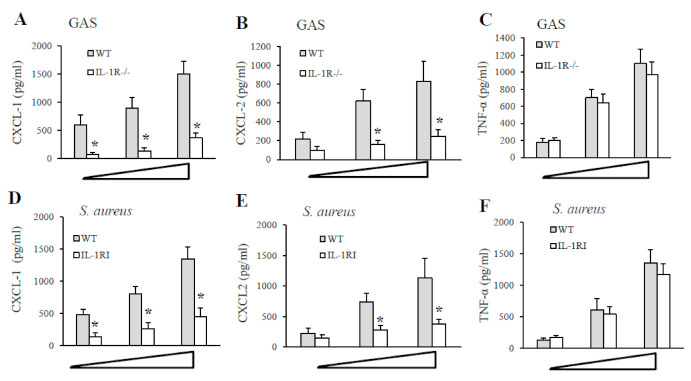
**Cytokines production by peritoneal macrophages stimulated with live GAS or *S. aureus*.** CXCL1, CXCL2 and TNF-α protein concentrations were measured by ELISA in culture supernatants of resident peritoneal macrophages from WT and IL-1R-deficient mice at 24 h after infection with increasing MOIs (1, 5, and 10) of GAS (**A**–**C**) or *S. aureus* (**D**–**F**). Data are expressed as means ± standard deviations of three duplicate observations, each from a different experiment. *, *p* < 0.05 relative to WT mice by one-way analysis of variance and the Student–Keuls–Newman test.

As shown in Figure 4, resident peritoneal macrophages from IL-1R-deficient mice or WT mice were stimulated with different multiplicities of infection (MOI; 1, 5 and 10) of GAS or *S. aureus* strain, and supernatants were collected after 24 h for cytokine assays. A significant reduction in CXCL1 and CLCL2 production was found in supernatants from IL-1R-deficient macrophages after GAS or *S. aureus* stimulation compared with WT cells (Figure 4, panels A, B, D and E). In contrast, no significant difference in the TNF-α levels was detected in the two experimental groups both using GAS or *S. aureus* as a stimulus (Figure 4, panels C and F). Similar data were obtained using heat-killed instead of live bacteria (data not shown). These data indicate that IL-1R signaling plays an important role in both GAS and *S. aureus*-induced production of remarkable CXCL1 and CXCL2 levels.

### 2.5. The Lack of IL-1 Signaling Is Associated with Reduced Neutrophil Recruitment at Sites of GAS and S. aureus Infection

**Figure 5:**
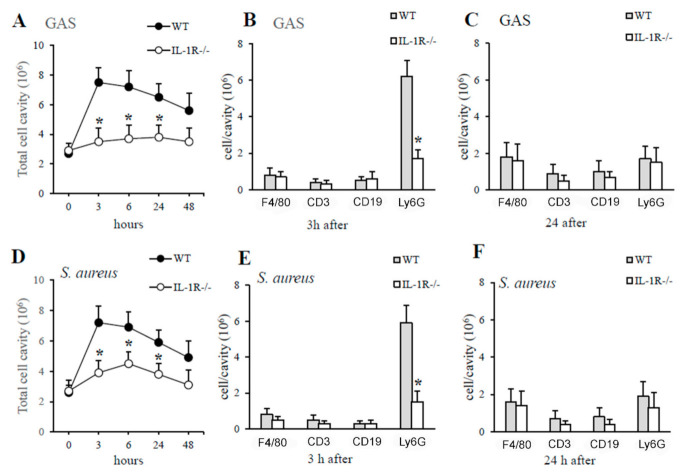
IL-1R deficiency is associated with impaired neutrophil recruitment in GAS- or *S. aureus*-induced peritoneal exudates. Kinetics of cell recruitment in the peritoneal cavities of IL-1R-deficient and WT mice after a challenge with killed GAS (**A**) or *S. aureus* (**D**). Numbers of peritoneal cells positive for F4/80 (macrophages), CD3 (T lymphocytes), CD19 (B lymphocytes), and Ly6G (neutrophils) in WT and IL-1R-deficient mice at 3 h (**B**,**E**) and 24 h (**C**,**F**), after a challenge with heat-killed GAS (**A**–**C**) or *S. aureus* (**D**–**F**). Data are expressed as means ± standard deviations of three duplicate observations, each conducted with a different animal, in the course of a single experiment. *, *p* < 0.05 relative to WT mice by one-way analysis of variance and the Student–Keuls–Newman test.

Based on the above data, it was of interest to determine whether IL-1 signaling is essential for optimal production of the neutrophil chemokines CXCL1 and CXCL2 after GAS or *S. aureus* challenge and, therefore, whether IL-1R-deficient mice would display reduced neutrophil recruitment at sites of GAS or *S. aureus* infection. In preliminary experiments, we determined the number and types of cells present in the peritoneal cavities of mice under basal conditions and, as previously reported [32], they were similar in the knockout and wild-type mice (data not shown). In particular, the percentage of neutrophils was less than 1% out of a total of just under 3 million peritoneal cells per cavity in both IL-1R-deficient and WT mice (data not shown). Thus, the role of IL-1-signaling in neutrophil recruitment was investigated by the injection of killed bacteria in the peritoneal cavity of WT and IL-1R-deficient mice. We did not use live bacteria because the results obtained were not reproducible due to uncontrolled bacterial growth in IL-1-deficient mice, as indicated by our previous experiments (data not shown). Next, mice were inoculated i.p. with killed GAS or *S. aureus* and peritoneal lavage fluid was obtained at different times after challenge. As shown in Figure 5 the kinetics of cell influx was similar for both types of bacteria but was deeply different in both groups of animals (WT and IL-1R-deficient mice). In particular, the curve that describes cell influx into the peritoneal cavities of WT mice rose rapidly after challenge, peaking at 3 h and remaining at levels above the baseline for at least 24 h (Figure 5). In contrast, the curve that describes cell influx into the peritoneal cavities of IL-1R-deficient mice showed a significant decrease in total cell count after stimulation, due to a selective decrease in neutrophil influx at early times after challenge (Figure 5).

### 2.6. The Lack of IL-1 Signaling Is Associated with Decreased Production of Neutrophil Chemokines and Neutrophil Influx during GBS Infection

**Figure 6:**
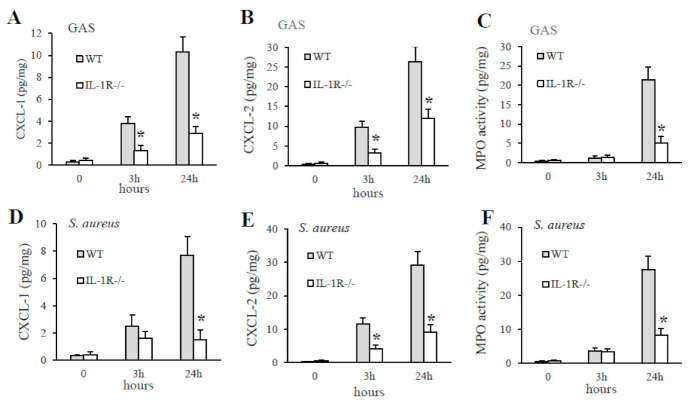
The lack of IL-1R signaling causes a serious deficiency in the production of CXCL1/2 chemokines and the subsequent recruitment of neutrophils to GAS or *S. aureus* infection sites. CXCL-1, CXCL-2, and MPO protein levels were measured in brain homogenates from WT and IL-1R deficient mice at the indicated times after i.v. infection with 1 × 10^6^ CFU of GAS (**A**–**C**) or 1.5 × 10^6^ CFU of *S. aureus* (**D**–**F**). Data are expressed as means ± standard deviations of three duplicate observations, each conducted with a different animal, in the course of a single experiment. *, *p* < 0.05 versus WT mice, as determined by one-way analysis of variance and the Student–Keuls–Newman test.

As detailed above, using a model of challenge with killed GAS or *S. aureus*, IL-1-deficient mice showed a severe defect in CXCL1/2 production and impaired recruitment of neutrophils to peripheral sites of infection. To investigate whether these effects were also evident during GAS or *S. aureus* infection, we infected mice by the i.v. route with live bacteria and collected their brains after different times. As shown in Figure 6, the organs of IL-1R-deficient mice infected with GAS or *S. aureus* had significantly lower CXCL1 and CXCL2 protein levels than those of WT mice. Moreover, significantly lower concentrations of myeloperoxidase (MPO), an index of neutrophils infiltration into the injured tissue, were detected in the brains of IL-1R deficient mice after infection with GAS or *S. aureus* (Figure 6, panel C and F).

## 3. Discussion

Having previously demonstrated that IL-1 signaling has a crucial role in host defenses against GBS [32], in the present study, we sought to investigate whether IL-1 signaling through the IL-1 receptor plays a crucial role also against two other frequent human Gram-positive pathogens, such as GAS and *S. aureus*. To this end, we set up two novel experimental models whereby leukocytes are recruited to the peritoneal cavity after challenge with GAS or *S. aureus*. We initially found that mice lacking IL-1R showed decreased abilities to control and thereby prevent subsequent bacterial spreading into the bloodstream. Moreover, data collected from the i.v. models indicated that IL-1 signaling stimulates anti-GAS or anti-*S. aureus* host defenses to restrict bacterial spreading from the initial site of infection and extend to these pathogens the results of our previous work on the importance of IL-1R in host defenses against Gram-positive bacterial pathogens [32].

Analyzing the data obtained from the i.v. and i.p. models together, the inability of IL-1R-deficient animals to control systemic bacterial spreading from the initial site of infection is clearly indicated.

Next, in light of these findings, we sought to determine whether the inability of IL-1R-deficient animals to clear an infection could be related to impaired phagocytosis and bacterial killing by phagocytes. As shown in Figure S1 and expected by previously data obtained using as stimulus GBS [32], no differences in the phagocytosis and killing of GAS or *S. aureus* were found between WT and IL-1R-deficient cells. Since we previously showed that IL-1 signaling is crucial for early neutrophil recruitment at peripheral sites of GBS infection [32], in further experiments, we sought to investigate whether such a phenomenon was also present in response to GAS or *S. aureus*. The results of these experiments clearly indicate that an early increase in neutrophil counts, in the peritoneal cavity, was detected in WT, but not in IL-1R-deficient mice, in response to killed GAS or *S. aureus*. Only 24 h later, a moderate increase in macrophage numbers was observed in both groups of animals (data not shown). Taken together, these data clearly indicate an important role for IL-1 signaling in early neutrophil, but not macrophage, influx in response to GAS or *S. aureus*. Having previous studies showed that the lack of IL-1 signaling is linked with decreased production of the chemokines CXCL1 and CXCL2 and neutrophil recruitment to GBS infection site, in further experiments we investigated is these effects were also evident with these two other species of extracellular bacteria [18]. The data presented here indicate that L-1R signaling is essential for CXCL1 and CXCL2 production upon exposure to GAS and *S. aures* and for neutrophil recruitment in a major target organ during bacterial infection.

These findings extend those of previous studies on the importance of IL-1R in host defenses against extracellular bacteria, such as *Streptococcus pneumoniae* and *S. aureus* [26,29,30,31,33]. Moreover, IL-1 signaling plays an important role in the host responses against different microorganisms such as: intracellular microorganisms (*Francisella tularensis, Listeria monocytogenes* and *Leishmania major*), virus (mouse adenovirus type 1) and fungi (*Trichophyton rubrum*). Collectively, the data presented here demonstrate a mechanism whereby the production of IL-1 induced by GAS or *S. aureus* stimulates the macrophages to produce chemokines involved in the recruitment of neutrophils. However, these data could not exclude that other cells, in addition to macrophages, can contribute to CXCL1 and CXCL2 production. Since IL-1R mediates signaling initiated by IL-1α or IL-1β [19], further studies are needed to identify which among these two ligands mediates early chemokine production and neutrophil recruitment during GAS or *S. aureus* infection. However, it is most likely the release of IL-1β, rather than IL-1α, associated with chemokine induction and neutrophil recruitment during GAS or *S. aureus* infection, as previously reported in other infection models involving bacterial pathogens [34,35]. To our knowledge, this is the first demonstration of the importance of IL-1 signaling in the host defenses against GAS- or *S. aureus* peritonitis in mice. However, further studies are needed to clarify the role of IL-1 signaling in multiple types of GAS or S. aureus infections since as recently showed by La Rock et al. neutrophil influx promoted by IL-1 signaling in response to GAS infection has an opposite role, protective or detrimental, depending on site of infection [25]. Together, our previous studies and the present one clearly show that IL-1R-dependent neutrophil recruitment to the peritoneal site of infection by major extracellular Gram-positive bacteria is absolutely required for the clearance of these bacteria by host innate immune defenses.

In conclusion, our data show that IL-1-dependent signaling has a fundamental role in the innate host defense against GAS or S. aureus, two extracellular deadly pathogens. The crucial role of IL-1signaling that we observed in this mouse model may also be important in human patients as evidenced by the administration of IL-1 receptor antagonist (anikara) to patients with active infections [19]. Further research is required to determine whether stimulation of IL-1 signaling may be useful as an alternative approach to treat GAS or *S. aureus* infections.

## 4. Materials and Methods

### 4.1. Mice, Reagents and Bacterial Strains

IL-1R-defective mice (Jackson Laboratories) and control WT C57BL/6 mice were purchased from Charles River Italia. The mice (7-wk old) were housed under pathogen-free conditions in enclosed filter-top cages at the Dipartimento di Human Pathology of the University of Messina (University of Messina, Messina, Italy). *Streptococcus pyogenes* (strain 3348) and *Staphylococcus aureus* (strain Newman) were provided by GSK vaccines srl, Siena, Italy. Unless otherwise specified, all reagents were from SigmaAldrich.

### 4.2. Murine Infection Models

Seven-week-old female mice were injected i.p. or i.v. with the indicated doses of the *Staphylococcus aureus* or GAS, as previously described [18]. Briefly, bacteria were grown to the mid-log phase in Todd–Hewitt (TH) broth (Oxoid) and diluted to the appropriate concentration in phosphate-buffered saline (PBS; 0.01 M phosphate, 0.15 M NaCl, pH 7.2) before inoculation of animals. In each experiment, the actual number of injected bacteria was determined by colony counting. Mice were observed every 12 h for 10 days after inoculation. In selected experiments, mice were sacrificed at the times indicated to measure bacterial burdens and plasma cytokine levels in the peritoneal lavage fluid, blood, and/or brain. In the experimental models of bacteria-induced inflammation, mice were injected with heat-killed *S. aureus* or GAS (0.5 mg) in PBS (0.2 mL) and peritoneal lavage fluid was collected at the times indicated to measure cell numbers by flow cytometry and cytokine concentrations (see below). Heat-killed, lyophilized bacteria were prepared by heat treatment (80 °C for 45 min), followed by extensive washing with distilled water and lyophilization exactly as previously described [18].

### 4.3. Organ Homogenates

Organ homogenates were collected exactly as previously described [18,32]. Briefly, brains were placed in preweighed sterile tubes (M-tubes; Miltenyi Biotech) containing PBS, and homogenized in the gentle MACS system (Miltenyi Biotech). Serial dilutions were prepared in duplicate and plated on blood agar for colony counting. Cytokines and intracellular MPO levels were determined in the homogenates as described below.

### 4.4. Cytokine and MPO Measurement

TNF-α, CXCL1, CXCL2, IL-6, and MPO concentrations were determined in duplicate with Duoset TNF-α, CXCL2/MP1 Quantikine, CXCL1/KC Quantikine, IL-6 Quantikine, and Myeloperoxidase DuoSet murine enzyme-linked immunosorbent assay (ELISA) kits according to the manufacturer’s recommendations (R&D Systems). The lower detection limits of these assays were, respectively, 16, 8, 16, 8, and 250 pg/mL. In preliminary experiments, a close correlation was found between intracellular MPO concentrations and neutrophil numbers, exactly as previously reported [18,32].

### 4.5. Flow Cytometry

Flow cytometry analysis of leukocyte subsets in peritoneal lavage fluid was performed on a FACS Canto II flow cytometer (BD Biosciences) as previously described [18,32]. Briefly, cells were resuspended at 1 × 10^6^ cells/100 µL in PBS and incubated with 0.5 μg Fc Block (BD Biosciences) for 20 min at 4° C. After incubation, fluorescently-labeled anti-F4/80 (macrophages), anti-CD3 (T lymphocytes), anti-CD19 (B lymphocytes), and anti-Ly-6G (neutrophils) were added and plates were incubated for 20 minutes at 4 °C in the dark, using the respective isotype Abs as controls. After incubation, cells were washed three times with FACS buffer. Cell counts were determined using BD TruCount tubes, as previously described [18,32]. Debris was excluded in a forward/side scatter (FSC/SSC) dot plot. Samples were analyzed on a FAC S Canto II flow cytometer with the FlowJo software, as previously described [32]. All of the reagents used for flow cytometry were from BD Biosciences.

### 4.6. Growth Curves of Bacteria in Whole Blood

*S. aureus* or GAS were grown in TH broth to the late exponential phase and harvested by centrifugation at 5000 g for 10 min, as previously described [32,36]. Briefly, freshly collected heparinized whole blood (0.2 mL) from WT or IL-1R-deficient mice was inoculated with 2.5 × 10^4^ of, respectively, GAS or *S. aureus* strain. Bacterial CFU was enumerated at the indicated time points after incubation at 37 °C.

### 4.7. Macrophage Phagocytosis and Killing Assays

Bone marrow-derived macrophages were prepared by flushing femurs and tibiae as previously described [18,37]. Briefly, the cells, after centrifugation, were resuspended to a concentration of 2.5 × 10^6^/mL and cultured for 7 days in a medium supplemented with 100 ng/mL of macrophage colony-stimulating factor (M-CSF; Peprotech) to obtain macrophages. Every 3 days, half of the medium was replaced with fresh, cytokine-supplemented culture medium. Cells cultured in M-CSF were found to be greater than 96% positive for CD11b, greater than 88% positive for F4/80, and less than 4% positive for CD11c by flow cytometric analysis. For phagocytosis assays, 2 × 10^6^ CFU of GAS or *S. aureus* strain were added to 5 × 10^5^ bone marrow-derived macrophages and incubated for 1 h. After washing three times with PBS was added medium containing penicillin (5 µg/mL) and gentamicin (100 µg/mL) and incubation for 1 or 2 h to kill extracellular bacteria. Bacterial CFU was enumerated by serial plating on TH agar plates after lysing cells with 0.025% Triton X-100 to release intracellular bacteria, as previously described [18]. For bacterial killing assays, 1 × 10^5^ CFU of GAS or *S. aureus* strain were added to 5 × 10^5^ macrophages for 2 h, followed by the addition of 50 µL of Triton X-100 solution to lyse cells. Recovered bacteria were plated on TH agar plates for CFU enumeration.

### 4.8. Stimulation of Peritoneal Macrophages

Resident mouse peritoneal macrophages were isolated from the peritoneal cavity by washing with ice-cold PBS as previously described [18]. Briefly, the cells collected by centrifugation and resuspended in RPMI 1640 (Euroclone) supplemented with 5% heat-inactivated fetal calf serum, 50 IU of penicillin/mL, and 50 g/mL of streptomycin, were seeded into the wells of 96-well dishes at a density of 5 × 10^5^/well and incubated at 37 °C in a 5% humidified CO^2^ environment. After removing non-adherent cells, adherent cells were stimulated at 37 °C for 30 min with increasing multiplicities of infection (MOIs; 1, 5, and 10) of GAS or *S. aureus*, as previously described [18]. The number of viable bacteria used in each experiment was carefully determined by plate counting. After incubation, the monolayers were incubated for 18 h in the presence of penicillin (250 IU/mL) and streptomycin (250 µg/mL) to limit the growth of residual extracellular bacteria. In selected experiments, cells were stimulated with increasing doses of heat-killed GAS or *S. aureus* (1, 5, and 10 µg/mL). Cell culture supernatants were collected at 18 h after stimulation to measure cytokine levels.

### 4.9. Data Expression and Statistical Significance

Differences in cytokine levels and organ CFU counts were assessed by one-way analysis of variance and the Student–Keuls–Newman test. Survival data were analyzed with Kaplan–Meier survival plots, followed by the log-rank test (JMP Software; SAS Institute, Cary, NC, USA) on an Apple Macintosh computer. When *p* values of less than 0.05 were obtained, differences were considered statistically significant.

## Data Availability

Not applicable.

## Supplementary Materials

The following are available online at https://www.mdpi.com/2076-0817/10/2/132/s1.
