# Supplementary material for: The Relevance of IL-1-Signaling in the Protection against Gram-Positive Bacteria
Source: Pathogens. 2021 Jan 28;10(2):132. doi: 10.3390/pathogens10020132 (PMC7911888; doi:10.3390/pathogens10020132)
Supplement: Supplementary file 1 [file pathogens-10-00132-s001.pdf]

Fig. S1

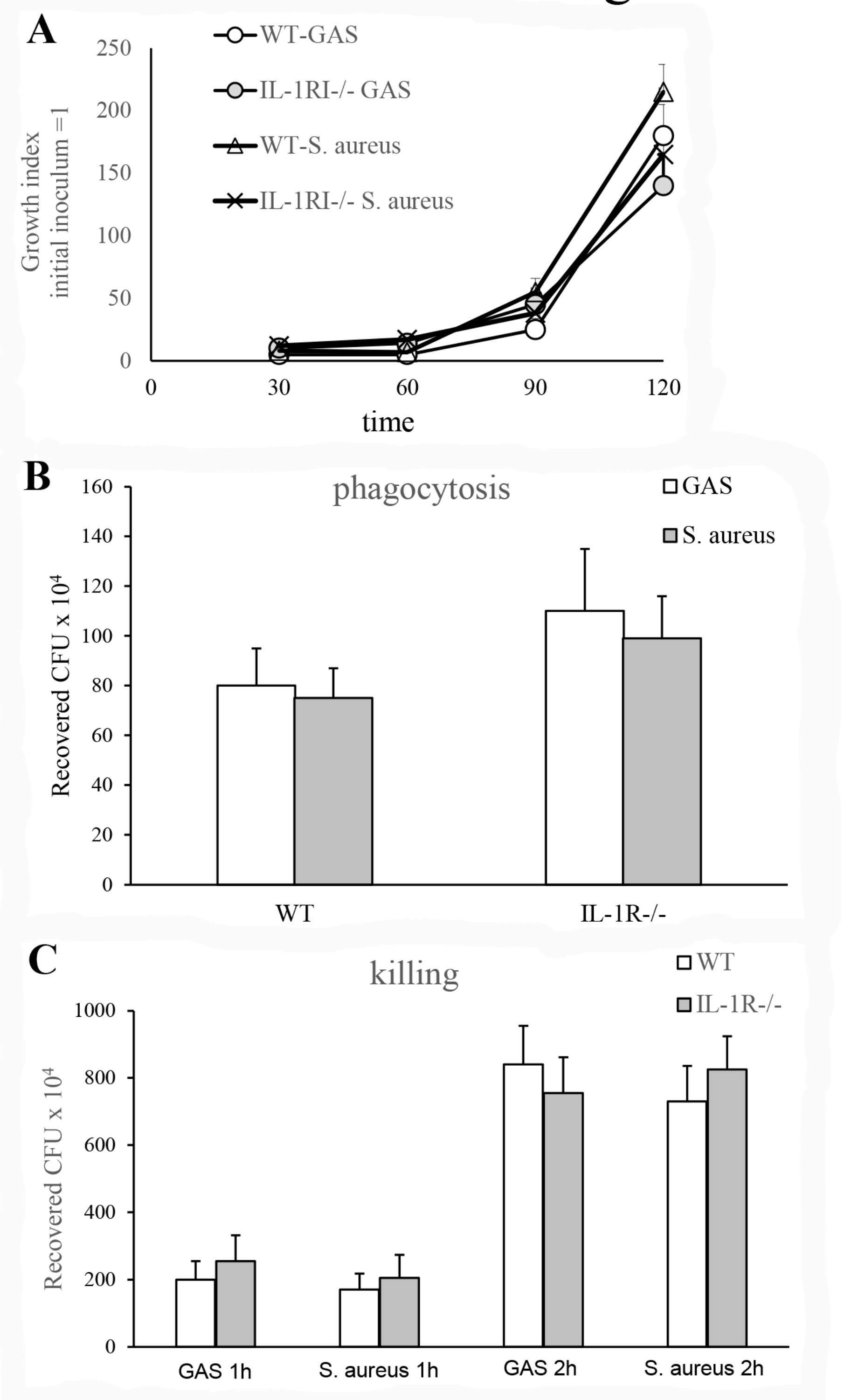

Figure S1. IL-1 signaling has no effect on phagocytosis and killing of GAS or S. aureus. (A), Whole blood (0.2 ml) from WT or IL-1RI KO mice was infected with 2 x 104 GAS or S. aureus and bacteria were enumerated at the time points indicated after incubation at 37°C. Phagocytosis (B) and killing (C) of GAS or S. aureus by bone marrow derived macrophages from WT and IL-1RI-deficient mice are also shown. Data are expressed as means ± standard deviations of triplicate observations, each from a different experiment.
